# Fourteen Letters and Replies

**Published:** 1875-04

**Authors:** 


### Letters Exposing Humbugs and Swindlers,

**With other Communications of Interest to the People.**

To DR. J., *Wellsboro, Pa.*—Boerick & Tafel, No. 145 Grand street, Phila., publish the work referred to.

---

To DR. R., *Pittsburg, Pa.*—Write to Geo. Tiemann & Co. 67 Chatham street, New York, for their catalogue of Surgical Instruments. You will find there what you need.

---

MILROY, PA., Jan. 2, 1875.

BISTOURY:

We like you very much. Have a good many laughs when reading you. We have been getting you for some time, and have some notion of having you bound into a volume when we get enough to make a good sized one. Have you any bound numbers? Yours,

A. L. H., M. D.

We have the BISTOURY bound in two handsome volumes, of 324 pages each, with copious index, price $2.50 per volume.

---

GENEVA, N. Y., March 1, 1875.

THAD S. UP DE GRAFF, M. D.:

I see an illustration of *opacity of the cornea*, in last number of the BISTOURY. You do not say whether such films can be removed. My left eye has had precisely such a spot as you describe, upon it, caused by severe inflammation of the eyes when a child. It covers the entire clear portion of the eye, but is not painful or inflamed. Can the film be removed?

S. A. G.

No.

---

CAROLINE, N. Y., March 20, 1875.

DR. UP DE GRAFF:

I saw old Major ——, whom you operated upon for *cataract* last winter, he has been trying to persuade me to come for an operation too. I had one eye operated upon at ———, and lost it from inflammation. Will that circumstance make it dangerous to try the remaining eye?

B.

The loss of one eye will in no way increase the risk upon the remaining one. Your chances for a successful operation are just as good as though the accident had not occurred.

LOUISVILLE, KY., March 18, 1875.

DR. UP DE GRAFF:

I see from the BISTOURY that you answer questions: Will you inform me whether "Sage's Catarrh Remedy" is of any worth as a curative agent?

J. S. P.

We cannot recommend *any* patent medicine as a curative. The one you refer to is as good as any. Better consult your physician than attempt to prescribe for yourself.

---

BROWNSVILLE, Tenn., January 20, 1875.

Dr. UP DE GRAFF:—Since you are so kind as to answer questions, I write to know if there is anything that will remove the enlargement of the bone at the joint of the toe. It is not sore, but gradually increases in size, so as to disfigure the foot. Please be so kind as to relieve one who is a constant reader and admirer of the "BISTOURY."

R. C. N.

You will find an answer in the article upon deformities of the toes, in the present number, with an illustration of the apparatus needed.

---

DR. THAD S. UP DE GRAFF:

I notice in last BISTOURY, an article on opacity of the cornea. Is there anything that can be put in the eye that will remove such an opacity, supposing there is little or no inflammation, and the opacity of from one to ten years standing. The treatment of this disease has been, with me, always very unsatisfactory. The best treatment in next BISTOURY will oblige, Yours truly,

J. A. H., M. D.

Opacities of the cornea, depending upon changes in the epithelial layer, the result of cornitis, granular lids, and superficial ulcers of the cornea, are treated with the following ointment:

℞.

Iodide of potassium, gr. j.

Yellow oxide of mercury, gr. ij.

Adipis, ʒ j—ʒ ij. M.

Of this, place a piece the size of a kernel of wheat, between the eyelids, twice daily.

We often inject beneath the conjunctiva, and as near to the opacity as possible, two or three drops of the following solution, with a hypodermic syringe:

℞.

Chloride of sodium, ℈ j.

Distilled water, ℥ j.

The solution should be filtered and warm. After the injection apply a compress bandage. This may be done once or twice a week.

JAMESTOWN, N. Y., March 19, 1875.

DR. UP DE GRAFF:

Will you please inform me in the April No. of the BISTOURY whether such an apparatus as you describe and illustrate in the January BISTOURY, for club foot, on page 322, will cure a child's foot, under one year of age, without cutting? I have a child so afflicted, and I prefer to have it cured without cutting, of course, if I can.

J. L. S.

It is possible that the apparatus referred to, will effect the cure without surgical interference. A surgeon would be the best judge of that. Better bring the child here for examination, when proper advice can be given.

---

TOWANDA, PA., February 16, 1875.

DR. UP DE GRAFF:

I have a little boy, aged 10, who, one year ago, injured his right eye in exploding percussion caps, a particle of the cap lodging in his eye and destroyed the vision. The eye has never fully recovered from the accident, but remains painful, and at times seriously injures his well eye, compelling him to shun the light. We fear a portion of the cap remains in his eye. What would you advise under the circumstances?

J.

The remedy in this case, is *extirpation* of the eye. The earlier it is removed, the better. The eye being blind and painful, is of no further use, but is an absolute and dangerous irritant to the sound one. Have the eye removed, by all means.

---

BANGOR, MAINE, Feb. 20, 1875.

DEAR DOCTOR:

I see you are in the habit of prescribing through the BISTOURY, for all manner of ailments; can you tell what is "good for" a tired and used up doctor? It may be very pleasant for you to sit in your cozy office, in the "Elmira Surgical Institute," and prescribe for those who come to you, but it is entirely a different matter for the doctor to go to his patients, riding over hill and dale, day and night. I repeat, what can you prescribe for the tired doctor?

L. B., M. D.

Of course, when doctors prescribe for each other, it must be done in a thoroughly orthodox manner. If this prescription were given in English, everybody would recognize it at once, and there is no telling how many we might induce to try it, for the very wording of it would indicate its palatableness. Taken in the Latin form, temperance folks could indulge in a "gin cocktail," without being aware of it.

℞. Spiritus Hordie et secalis cum lupulina destilati, et cum baccis juniperi communis redestillate et rectificati, f. ℥ j.

Tincturæ gentianæ et amomi cardamomi compositæ, f. ℥ ss; Sacchari albi cochleare, minimum unum.

Aquæ frigidæ, f. ℥ iij. Misce bene cum fustula, et adde: Corticis limonis recentis, sectionem parvulam. Signa. Quater die hauriendum. Videlicet: Mane, una hora post meridiem, ad vesperam, et ad horam recumbendi.

---

LEROY, Pa., March 24, 1875.

DEAR DOCTOR:

Why don't you make your BISTOURY a monthly journal? I, in common with many others I know of, would willingly pay $1.50 or $2.00 per year for it, if it came to us monthly,

J. S. B., M. D.

Our only reason for not complying with the wishes of our friends upon this subject is, a want of time. Our professional duties are such as to absolutely forbid the undertaking.

---

CUBA, N. Y., Jan. 30, 1875.

DR. UP DE GRAFF:

Some one has sent me a copy of the BISTOURY, for January, in which I find an illustrated article upon the cure of cleft-palate. I was not aware before, that this trouble was curable. I have a child, six years old, with a cleft through the entire roof of her mouth, so that she speaks very indistinctly. Is she too young for such an operation as you describe, or would you recommend it to be done now.

L. B.

Have the operation performed now. It is much easier to accomplish while the parts are yielding, as in a child of her age.

---

HISEVILLE, KY., March 9, 1875.

THAD. S. UP DE GRAFF, M. D.:

DEAR SIR—I have taken the BISTOURY, and paid for it, from its infancy. I can't do without it. I find in no other medical journal the same amount of useful and practical knowledge. I wish it was a weekly, instead of a quarterly journal. Sound to the core, fearless in the abuse of error, and prompt in the punishment of Charlatanism; I hope it will live long with its shining light, and that the editor may reap a rich harvest of reward in this life, and a more glorious one in the future beyond. Very truly, yours,

T. L. NEWBERRY, M. D.

Thanks, Doctor, imagine that our bow has been duly made and recorded.

